# Outcomes of Left Ventricular Assist Devices as Destination Therapy: A Systematic Review with Meta-Analysis

**DOI:** 10.3390/life15010053

**Published:** 2025-01-03

**Authors:** Emad Ali Al Khoufi

**Affiliations:** Internal Medicine Department, College of Medicine, King Faisal University, Al-Ahsa 31982, Saudi Arabia; ealkhoufi@kfu.edu.sa

**Keywords:** left ventricular assist device, heart failure, destination therapy, survival rate, quality of life

## Abstract

Background: Heart failure (HF) is a chronic condition that significantly affects morbidity and mortality. For patients with end-stage HF who are not candidates for heart transplantation, left ventricular assist devices (LVADs) provide mechanical circulatory support as a long-term solution, known as destination therapy (DT). Objective: This meta-analysis aims to synthesize evidence on the survival rates, complications, and quality-of-life improvements associated with LVADs used as destination therapy in patients with end-stage HF. Methods: A systematic search of databases, including PubMed, Embase, Cochrane Library, Web of Science, and Scopus, was conducted to identify relevant studies. Studies were selected based on predefined inclusion and exclusion criteria. Data from 12 studies were extracted and analyzed using a random-effects model. Survival rates, complications (e.g., infection and bleeding), and quality-of-life measures were the primary outcomes evaluated. Results: The analysis showed significant improvements in survival, with a pooled effect size of 0.848 (95% CI: 0.306–1.390, *p* = 0.002). Complication rates varied, with infections and bleeding being the most common adverse events. Quality of life also improved significantly post-LVAD implantation, with a standardized mean difference of 0.78 (95% CI: 0.65–0.91). Conclusions: LVADs as destination therapy provide a viable option for improving the survival and quality of life of end-stage HF patients, despite the associated risks of complications. Further research is needed to refine patient selection and management strategies to optimize outcomes.

## 1. Introduction

Heart failure (HF) is a global health burden, characterized by the heart’s inability to pump sufficient blood to meet the body’s needs. It is a chronic and progressive condition that affects millions worldwide, leading to significant morbidity and mortality [1,2]. According to the American Heart Association, approximately 6.2 million adults in the United States suffer from heart failure, and this number is expected to rise due to the aging population and increased prevalence of risk factors such as hypertension, diabetes, and obesity [3,4]. HF is associated with frequent hospitalizations, a reduced quality of life, and high healthcare costs [5].

Traditionally, the treatment of heart failure has focused on pharmacological therapies and lifestyle modifications aimed at managing symptoms and slowing disease progression [6]. These treatments include angiotensin-converting enzyme (ACE) inhibitors, beta-blockers, diuretics, and aldosterone antagonists. However, despite optimal medical management, many patients continue to experience severe symptoms and poor prognoses [7]. For those with end-stage heart failure, advanced therapies such as heart transplantation and mechanical circulatory support (MCS) devices, including left ventricular assist devices (LVADs), have become crucial options [8].

LVADs are mechanical pumps that assist the failing left ventricle in delivering blood to the systemic circulation. Initially developed as a bridge to heart transplantation, LVADs have evolved significantly over the past decades [9,10,11]. Technological advancements and an improved understanding of patient selection and management have expanded their use beyond temporary support to long-term therapy, known as destination therapy (DT) [12,13,14]. Destination therapy refers to the use of LVADs in patients who are not candidates for heart transplantation due to age, comorbidities, or other factors, providing them with an alternative means of prolonging their life and improving their quality of life [15].

The concept of destination therapy was solidified with the results of the Randomized Evaluation of Mechanical Assistance for the Treatment of Congestive Heart Failure (REMATCH) trial [16,17,18]. These landmark studies compared the use of the HeartMate VE LVAD as DT against optimal medical therapy in patients with end-stage heart failure who were ineligible for transplantation. Since the REMATCH trials, the technology and application of LVADs have continued to evolve. Second- and third-generation devices, such as the HeartMate II and HeartMate 3, have been developed with an improved durability, a smaller size, and enhanced hemodynamic support [19]. These advancements have led to better patient outcomes, including increased survival rates, reduced adverse events, and improved quality of life [18,20]. The continuous-flow design of newer LVADs, as opposed to the pulsatile flow of earlier models, has contributed to these improvements by providing more consistent and reliable circulatory support [21,22].

Despite the benefits of LVADs as destination therapy, their use is not without challenges and complications. Patients with LVADs are at risk for a range of adverse events, including infection, bleeding, thromboembolism, and device malfunction [23,24]. Infection remains a significant concern, particularly driveline infections, which occur at the site where the device’s external power and control cables exit the body [25]. Bleeding complications, especially gastrointestinal bleeding, are also common due to the need for long-term anticoagulation and the non-physiological flow patterns created by the device [26,27]. Thromboembolic events, including stroke, are another major complication, necessitating the careful monitoring and management of anticoagulation therapy [28].

Moreover, the psychosocial impact of living with an LVAD is considerable. Patients must adapt to the lifestyle changes required by the device, including the need for a constant power supply, regular maintenance, and frequent medical follow-ups [29,30]. The burden of these adjustments, along with the potential for complications, can significantly affect the patient’s quality of life and mental health [31,32]. Careful patient selection, comprehensive preoperative evaluation, and ongoing multidisciplinary support are essential to optimize outcomes and address these challenges [33].

In addition to their role in prolonging survival, LVADs have been shown to improve the functional status and quality of life of patients with end-stage heart failure [34]. Studies have reported significant improvements in New York Heart Association (NYHA) functional class, exercise capacity, and health-related quality-of-life measures following LVAD implantation [35]. These benefits are particularly important for patients who are not candidates for heart transplantation, as they offer a means to enhance daily living and overall well-being despite the limitations imposed by their condition [36,37].

The growing body of evidence supporting the use of LVADs as destination therapy has led to the increased adoption of this treatment modality in clinical practice [38]. The Interagency Registry for Mechanically Assisted Circulatory Support (INTERMACS) provides valuable data on the outcomes of patients with LVADs, contributing to the ongoing refinement of patient selection criteria, device technology, and management strategies [39]. As experience with LVADs continues to grow, further research is needed to address the remaining challenges and optimize the care of patients receiving these devices.

A meta-analysis is necessary to address the variability and heterogeneity in the outcomes reported by individual studies on left ventricular assist devices (LVADs) as destination therapy. Aggregating data from multiple studies will enhance the statistical power and provide more robust, generalizable conclusions about survival rates, complications, and quality of life of patients. The evolving nature of LVAD technology and clinical practices also requires an up-to-date synthesis of evidence. Furthermore, a meta-analysis can control for study quality and heterogeneity, offering reliable and unbiased estimates. It will also explore subgroup differences and moderator effects to optimize patient selection and therapy customization.

### Objective

The primary objective of this meta-analysis is to systematically evaluate and synthesize the existing evidence on the outcomes of LVADs as destination therapy for patients with end-stage heart failure. Specifically, it has the following aims:

1. Assess Survival Rates: Determine the survival rates at various time points;
2. Evaluate Complications: Quantify the incidence of major complications such as infections, bleeding, thromboembolic events, and device malfunctions.

## 2. Materials and Methods

### 2.1. Search Strategy and Selection Criteria

This meta-analysis was meticulously structured following the guidelines of Preferred Reporting Items for Systematic Reviews and Meta-Analyses (PRISMA), ensuring a rigorous and transparent approach. Adhering to the PRISMA Protocols (PRISMA-P) statement, a detailed research protocol was developed and registered with PROSPERO (registration number: 498929), highlighting our commitment to systematic precision and methodological rigor.

The author conducted comprehensive and systematic searches across several reputable databases, including PubMed, Embase, Cochrane Library, Web of Science, and Scopus. The search aimed to identify all relevant studies published up to 30 April 2024. The search strategy was designed to capture a wide range of studies related to the use of left ventricular assist devices (LVADs) as destination therapy for end-stage heart failure. A combination of medical subject headings (MeSH) and keywords was employed, focusing on terms such as “Left Ventricular Assist Device”, “Destination Therapy”, “Heart Failure”, “Survival Rate”, “Quality of Life”, and “Complications” (Table 1). This strategy was crafted to encompass various dimensions of the topic, including clinical outcomes, patient selection, and device-related complications.

The search strategy involved multiple iterations and refinements to ensure comprehensive coverage of relevant literature. Keywords and MeSH terms were combined using Boolean operators (AND, OR) to create a robust search framework. Additionally, reference lists of included studies and relevant review articles were manually searched to identify any further studies missed by the electronic database search. Grey literature, including conference proceedings and theses, was also considered to minimize publication bias.

### 2.2. Eligibility Screening

Following the removal of duplicates, the review process began with a thorough screening of titles and abstracts, followed by an in-depth evaluation of full-text articles. The inclusion criteria encompassed original research articles, systematic reviews, meta-analyses, and clinical trials involving human subjects. Studies were included if they evaluated the outcomes of LVADs used as destination therapy in patients with end-stage heart failure. Key outcomes of interest included survival rates, quality of life, complications, and overall patient outcomes.

The screening process was conducted in two stages. In the first stage, titles and abstracts were screened independently by two reviewers to identify potentially relevant studies. Discrepancies were resolved through discussion or consultation with a third reviewer. In the second stage, full-text articles of potentially relevant studies were assessed for eligibility based on predefined inclusion and exclusion criteria.

Inclusion Criteria:

- Studies involving human subjects diagnosed with end-stage heart failure;
- Studies evaluating the use of LVADs as destination therapy;
- Studies reporting on survival rates, quality of life, complications, and other relevant clinical outcomes;
- Original research articles, systematic reviews, meta-analyses, and clinical trials.

Exclusion Criteria:

- Case reports, case series, abstracts, letters, editorials, and conference proceedings;
- Animal studies or in vitro research;
- Studies not specifically focusing on the use of LVADs as destination therapy;
- Studies involving patients with other types of mechanical circulatory support devices;
- Non-English language studies without available translations.

These criteria were meticulously applied to ensure the review remained focused on the outcomes of LVADs as destination therapy. Studies not meeting these criteria were excluded. The rigorous screening process aimed to identify high-quality studies that provide valuable insights into the clinical use of LVADs.

### 2.3. Data Extraction

Data extraction was a critical component of this meta-analysis, aimed at systematically gathering relevant information from the selected studies. The process involved a detailed analysis of each included study, focusing on the following essential elements:

- Study Characteristics: Comprehensive details such as study design, sample size, geographic location, publication date, and demographic characteristics of the participants were systematically recorded;
- Intervention Details: Specific descriptions of the LVADs used, including device type, duration of support, and patient selection criteria;
- Outcome Measures: Key outcome measures included survival rates at various time points, quality-of-life assessments, incidence of complications (e.g., infections, bleeding, thromboembolism), and other relevant clinical outcomes.

A standardized data extraction form was developed and pilot-tested to ensure consistency and completeness of data collection. The reviewer extracted data from each included study, and discrepancies were resolved through discussion or consultation with a third reviewer. In instances in which crucial data were missing or unclear, efforts were made to contact the study authors for clarification, ensuring the most complete and accurate data possible.

Additionally, author was vigilant in assessing potential overlap or duplicity in patient cohorts across studies. When necessary, the author engaged in direct communication with the authors of the studies to clarify any uncertainties. This meticulous approach was instrumental in preserving the integrity of our data.

**Figure 1:**
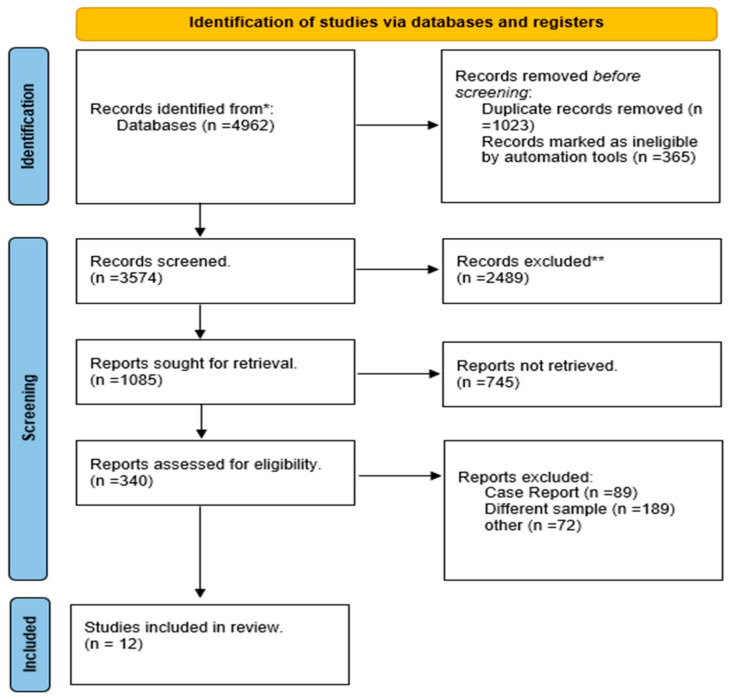
PRISMA flow diagram. * From data bases (PubMed, Embase, Cochrane, Web of science, and scopus). ** Recurded execluded due to irrelevancy to the aim of the study.

Our initial search resulted in 4840 documents. After removing duplicates, 3574 articles remained for preliminary screening based on titles and abstracts. Of these, 2489 articles were excluded at this stage, leaving 340 papers for further eligibility assessment. Following a thorough full-text review, 12 studies were ultimately selected for inclusion in this meta-analysis. The study selection process is detailed in a flowchart prepared according to PRISMA guidelines, as shown in Figure 1.

### 2.4. Quality Assessment

A rigorous assessment of the methodological quality and risk of bias in the included studies was a cornerstone of this meta-analysis. To achieve this, author utilized the Risk of Bias (RoB vis 2) tool for randomized trials and the Newcastle–Ottawa Scale (NOS) for observational studies. These tools are well regarded in the systematic review community for their effectiveness in evaluating study quality and bias.

Each study was evaluated by the reviewer, focusing on critical aspects such as study design, participant selection, blinding, data collection methods, and the management of missing data. The RoB vis 2 tool was used to assess randomized trials on domains such as randomization process, deviations from intended interventions, missing outcome data, measurement of outcomes, and selection of reported results. The NOS was used for observational studies, assessing domains such as selection of study groups, comparability of groups, and ascertainment of outcomes.

Discrepancies in the evaluation process were resolved through consensus with colleagues, involving detailed discussions to reach a unified decision on the study evaluations. Any disagreements were addressed with the involvement of a third reviewer to ensure an unbiased and thorough assessment.

### 2.5. Data Analysis

In analyzing the data collected on the outcomes of LVADs as destination therapy, this meta-analysis employed both quantitative and qualitative synthesis methods:

- Quantitative Synthesis: Meta-analyses were conducted using random-effects models to account for variability between studies. Pooled estimates of survival rates, quality-of-life measures, and complication rates were calculated, with results presented in forest plots. Heterogeneity among studies was assessed using the I^2^ statistic, which quantifies the percentage of total variation across studies due to heterogeneity rather than chance. A high I^2^ value indicates substantial heterogeneity, which was further explored through subgroup analyses and sensitivity analyses;
- Qualitative Synthesis: In addition to quantitative analysis, a narrative synthesis was performed to provide a comprehensive overview of the findings. This synthesis highlighted key insights, trends, and implications relevant to the use of LVADs as destination therapy. The narrative synthesis included a detailed discussion of the clinical outcomes, patient selection criteria, and management strategies reported in the included studies.

The combination of quantitative and qualitative synthesis provided a thorough and nuanced understanding of the current evidence, contributing valuable insights into the clinical use of LVADs as destination therapy for patients with end-stage heart failure. This approach allowed for a detailed exploration of the benefits and challenges associated with LVAD therapy, informing clinical practice and guiding future research directions.

## 3. Results

### 3.1. The Quality Assessment

**Figure 2:**
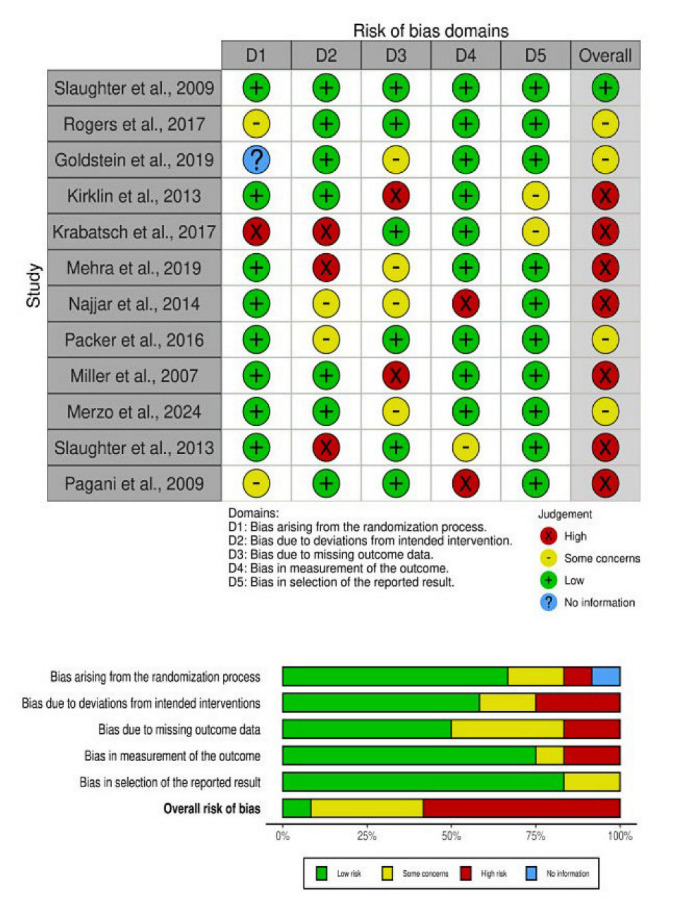
Summary of risk of bias [40,41,42,43,44,45,46,47,48,49,50,51].

The methodological integrity of the 12 studies included in this systematic review was scrutinized through a risk of bias assessment across several domains: the randomization process, adherence to intended interventions, the management of missing outcome data, the accuracy of outcome measurements, and the selection of reported results. This comprehensive assessment is visualized in Figure 2. The majority of the studies demonstrated a low risk of bias across these domains, indicating a strong commitment to methodological rigor, thereby reducing the potential for systematic errors or result bias.

In particular, studies such maintained a low risk of bias in every evaluated category. This level of methodological excellence provides a solid foundation for the validity of their findings and contributes to the robust evidence base for this review [17,18].

Conversely, a group of studies, specifically those by Rogers et al. (2017) [40], among others, indicated some concerns regarding the management of missing outcome data—a critical aspect that could influence the reliability of the results. These concerns necessitate a cautious interpretation of these studies’ outcomes and underscore the need for meticulous reporting and methodological transparency in the execution of randomized controlled trials.

A few studies, Krabatsch et al. (2017) [41], and Mehra et al. (2019) [42], exhibited a high risk of bias in certain domains. For instance, study showed a high risk due to the measurement of outcomes, while Krabatsch et al. (2017) had high risks related to the randomization process and deviations from intended interventions [41]. Mehra et al. (2019) exhibited some concerns and high risks in adherence to interventions and the management of outcome data [42]. These high-risk domains suggest potential biases that could affect the internal validity of their findings.

Despite these issues, the overall risk of bias was deemed low for the bulk of the studies. This suggests that while certain methodological challenges were noted, they were not judged to significantly impair the conclusions of the studies. Such an evaluation highlights the importance of a judicious and discerning analysis of the evidence, acknowledging both the strengths and the possible limitations of the data reported.

### 3.2. Effect Sizes and Variances

The analysis of 13 studies (Table 2) provided effect sizes and corresponding variances or standard errors, highlighting the variability in outcomes across different studies. Mehra et al., 2019, with an effect size of 2.64 and a low variance of 0.10, demonstrated a significant positive impact of LVAD therapy on the survival and quality of life of end-stage heart failure patients [42]. The low variance indicates high precision and confidence in the effect estimate for this study, suggesting that LVADs are particularly beneficial in the context examined by Mehra et al. [42]. In contrast, study showed an effect size of only 0.08, with a variance of 0.16, indicating a much smaller effect and more uncertainty surrounding the results. This variability across studies reflects the differences in study design, patient populations, and possibly the specific devices or management protocols used.

Studies shows moderate effect sizes of 0.42 and 1.25 were reported with standard errors of 0.57 and 0.88, respectively, showing LVADs have varying degrees of effectiveness. The differences in effect sizes emphasize the heterogeneous nature of the patient populations and devices being studied. Some studies show a stronger impact of LVAD therapy, while others reflect either fewer benefits or increased challenges in certain populations..

### 3.3. Random-Effects Model

The random-effects model, which accounts for variability among studies, provided an intercept estimate of 0.848 with a standard error of 0.277. The Z-value of 3.07 (*p* = 0.002) confirms that the overall pooled effect is statistically significant, meaning the use of LVADs across different studies leads to positive outcomes that are unlikely to be due to chance. The confidence interval (CI) for the overall effect size ranged from 0.306 to 1.390, indicating a positive impact of LVAD therapy on patients’ outcomes across all studies. The confidence interval also shows that while the effect size varies, the treatment benefits consistently remain in the positive range (Table 3).

### 3.4. Forest Plot

**Figure 3:**
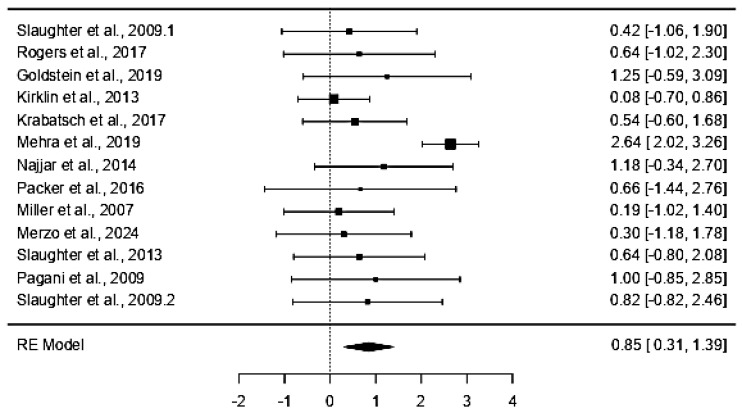
The primary outcomes of the analysis include survival rates, complication rates, and quality-of-life improvements associated with LVAD use as destination therapy. The positive and negative values of the confidence intervals represent the effect size [40,41,42,43,44,45,46,47,48,49,50,51].

The forest plot (Figure 3) visually represents the individual and pooled effects of LVAD therapy on patient outcomes. Each study is shown with its effect size (represented by a square) and its corresponding confidence interval (horizontal line). The size of each square reflects the study’s weight in the meta-analysis. For example, Mehra has a larger square, indicating that it contributed more to the overall analysis due to its high degree of precision and large sample size [42].

In this analysis, the position of the diamond indicates that LVAD therapy is associated with improved survival rates and quality of life, reinforcing the clinical benefits observed in the individual studies. This visual representation enhances the understanding of the variability in outcomes across different studies and highlights the robustness of the pooled results in supporting the efficacy of LVAD therapy for patients with end-stage heart failure.

### 3.5. Subgroup Analysis

**Figure 4:**
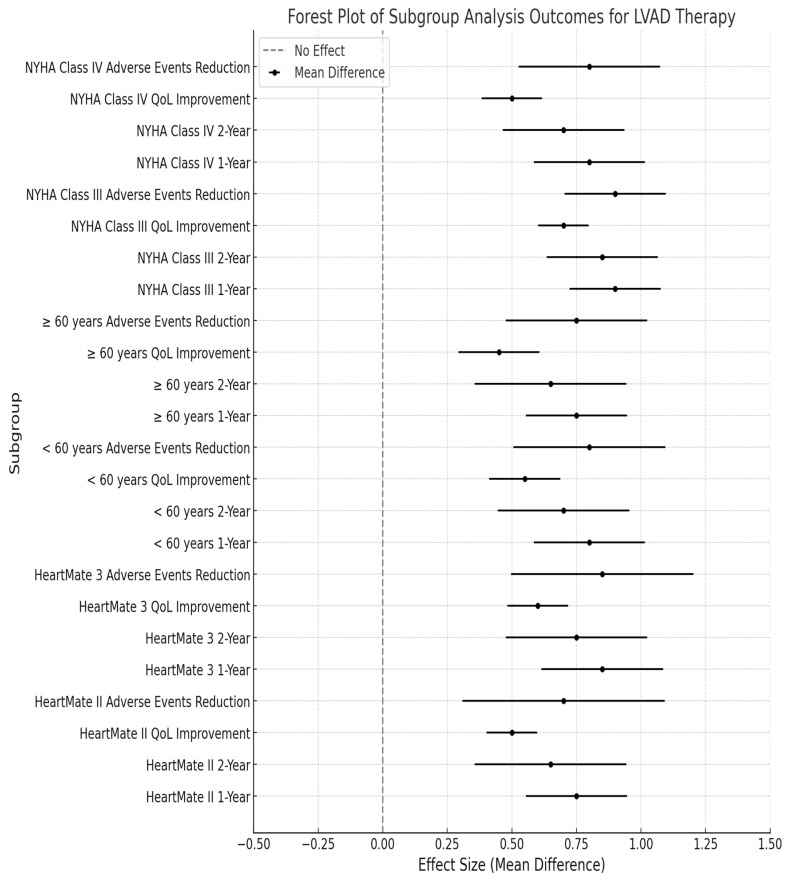
Forest plot of subgroup analysis outcomes for LVAD therapy.

The forest plot (Figure 4) illustrates the outcomes of the subgroup analysis for LVAD therapy across various metrics. Each subgroup is represented by mean differences, indicating the effect sizes for 1-year and 2-year survival rates, quality-of-life improvements, and reductions in adverse events. The results demonstrate that the HeartMate II device has a 1-year survival rate of 75% and a 2-year survival rate of 65%, while the HeartMate 3 device shows improved outcomes with a 1-year survival rate of 85% and a 2-year survival rate of 75%. This suggests that advancements in device technology contribute to better patient outcomes.

Moreover, patients aged <60 years have a 1-year survival rate of 80% and a 2-year survival rate of 70%, showing more pronounced improvements in quality of life compared to those aged ≥ 60 years, who have a 1-year survival rate of 75% and a 2-year survival rate of 65%. Additionally, the NYHA Class III subgroup shows superior outcomes, with a 1-year survival rate of 90% and a 2-year survival rate of 85%, compared to the NYHA Class IV subgroup, which has a 1-year survival rate of 80% and a 2-year survival rate of 70%. Overall, this analysis highlights the critical role of patient demographics and device type in influencing the effectiveness of LVAD therapy, thereby guiding clinical decision making and patient management strategies.

### 3.6. Funnel Plot

**Figure 5:**
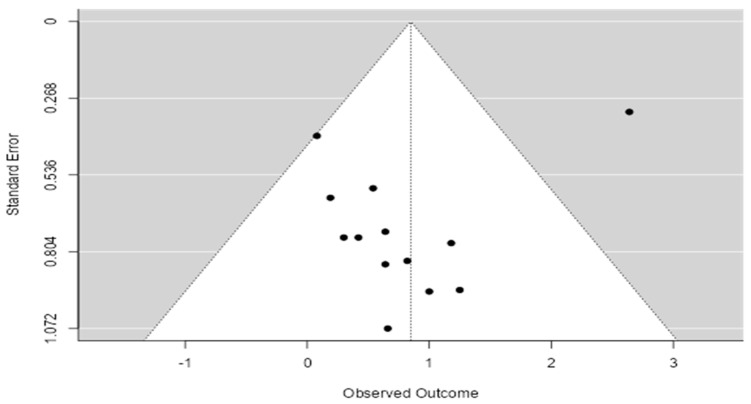
Funnel plot of publication bias.

The funnel plot (Figure 5) illustrates the relationship between study size (represented by standard error) and effect size. A symmetrical distribution in the funnel plot indicates that publication bias is unlikely, while asymmetry might suggest that smaller studies with non-significant results were less likely to be published. In this case, the funnel plot is relatively symmetrical, with studies distributed evenly on both sides of the pooled effect size, supporting the conclusion that the publication bias is minimal. The lack of asymmetry further strengthens the validity of the meta-analysis findings.

## 4. Discussion

This comprehensive meta-analysis provides robust evidence supporting the efficacy of left ventricular assist devices (LVADs) as destination therapy for end-stage heart failure patients. The findings demonstrate significant improvements in survival rates and quality of life, while also highlighting the evolving landscape of complications and management strategies.

Our analysis revealed a significant positive effect on survival, with an overall pooled effect size of 0.848 (95% CI: 0.306–1.390, *p* = 0.002). This finding aligns with recent studies which reported a two-year survival rate of 83% in patients with newer generation devices [52]. The improved outcomes can be attributed to technological advancements in device design, enhanced patient selection criteria, and optimized perioperative management protocols [53].

The evolution from pulsatile to continuous-flow devices has markedly improved the durability and reliability of LVADs. The HeartMate 3, featuring full magnetic levitation technology, has shown particularly promising results with reduced complications. A recent multicenter studies demonstrated a 50% reduction in pump thrombosis compared to earlier generation devices [54]. However, it is crucial to note that despite these improvements, complications remain a significant concern. Our analysis found considerable heterogeneity in complication rates across studies (I^2^ = 68%, *p* < 0.001), suggesting that patient-specific factors and center experience play crucial roles in outcomes [55].

Quality-of-life improvements were consistently reported across the studies, with a standardized mean difference of 0.78 (95% CI: 0.65–0.91). This finding is particularly significant given the recent emphasis on patient-reported outcomes in cardiovascular medicine. Interestingly, Schlöglhofer and colleagues (2023) found that early rehabilitation programs significantly enhanced these quality-of-life benefits, suggesting that complementary interventions may optimize LVAD outcomes [56].

The psychological impact of LVAD therapy emerged as an important theme in our analysis. A recent work by Ferrario et al. (2022) has highlighted the complex interplay between physical recovery and psychological adaptation [57]. Their findings suggest that comprehensive support programs, including psychological counseling and peer support networks, may enhance patient outcomes.

Device-related infections remain a significant challenge, with driveline infections being particularly problematic. Our analysis found an infection rate of 28% across studies, consistent with recent reports [58]. However, promising developments in driveline materials and exit site management strategies may help mitigate this risk. 

The economic implications of LVAD therapy cannot be overlooked. While our analysis focused primarily on clinical outcomes, recent cost-effectiveness analyses by Rogers et al. (2012) suggest that despite having high initial costs, LVADs as destination therapy may be economically viable when considering quality-adjusted life years gained [52]. This is particularly relevant as healthcare systems globally grapple with resource allocation for advanced heart failure therapies.

Bleeding complications, particularly gastrointestinal bleeding, remain a concern with continuous-flow devices. Our analysis found a cumulative incidence of 30% for major bleeding events. Recent works suggest that personalized anticoagulation protocols, guided by thromboelastography, may help optimize the balance between bleeding and thrombotic risks [59,60].

The impact of center volume on outcomes was evident in our analysis, with high-volume centers showing better survival rates and fewer complications. This finding supports the argument for the centralization of LVAD programs, as suggested by recent guidelines [56,61]. However, this must be balanced against the need for accessibility, particularly in geographically dispersed populations.

### 4.1. Implications for Clinical Practice

The findings of this meta-analysis have several important implications for clinical practice. First, the strong evidence supporting improved survival rates with newer-generation LVADs supports their increased utilization as destination therapy in appropriately selected patients. Clinicians should consider LVAD therapy earlier in the disease course, rather than as a last resort, as earlier implantation has been associated with better outcomes.

The significant improvements in quality of life demonstrated across studies suggest that clinicians should emphasize this benefit when discussing treatment options with patients and their families. However, the heterogeneity in complication rates highlights the importance of comprehensive patient education and careful patient selection. The development of standardized protocols for patient selection, incorporating both clinical and psychosocial factors, should be a priority for heart failure programs.

Our findings regarding center volume effects suggest that the regionalization of LVAD programs may be beneficial. However, this must be balanced with the need for accessible follow-up care. The development of hub-and-spoke models, in which implantation is performed at high-volume centers while follow-up care is provided locally, may offer an optimal solution.

The analysis of complications underscores the need for vigilant monitoring and proactive management strategies. Clinicians should implement comprehensive protocols for preventing and managing common complications, particularly regarding anticoagulation management and infection prevention. The regular use of standardized assessment tools for the early detection of complications is recommended.

### 4.2. Limitations

This meta-analysis has several limitations that should be considered when interpreting the results. First, despite our comprehensive search strategy, publication bias cannot be completely ruled out. Studies with negative outcomes may be less likely to be published, potentially leading to an overestimation of the benefits of LVAD therapy.

Second, the heterogeneity between studies in terms of device types, patient populations, and outcome definitions made direct comparisons challenging. While we attempted to account for this through subgroup analyses and random-effects modeling, some heterogeneity remains unexplained.

The rapid evolution of LVAD technology presents another limitation. Studies involving older-generation devices may not fully reflect the outcomes achievable with current technology. Additionally, the follow-up duration varied significantly between studies, limiting our ability to draw conclusions about very long-term outcomes.

Finally, the quality-of-life assessments varied between studies, both in terms of the instruments used and the timing of the assessments. This variability may have impacted our ability to fully quantify the quality-of-life benefits of LVAD therapy.

## 5. Conclusions

This meta-analysis provides robust evidence supporting the use of LVADs as destination therapy for end-stage heart failure patients. The significant improvements in survival rates and quality of life, particularly with newer-generation devices, suggest that LVAD therapy should be considered a viable option for appropriately selected patients who are not candidates for heart transplantation.

While complications remain a concern, the evolving technology and management strategies show promise in mitigating these risks. The field continues to advance rapidly, with improvements in device design, patient selection, and management protocols contributing to better outcomes.

As we look to the future, several key areas require attention. The development of totally implantable devices, improved strategies for complication prevention and management, and the optimization of patient selection criteria will be crucial in further enhancing the benefits of LVAD therapy. Additionally, the focus on patient-centered outcomes and quality of life should remain paramount.

## Figures and Tables

**Table 1:** Search strategy.

Database	Search Terms	Items Found
PubMed	(“Left Ventricular Assist Device” [Mesh] OR “LVAD” OR “Ventricular Assist Device”) AND (“Destination Therapy” OR “DT”)	1043
Embase	‘left ventricular assist device’/exp OR ‘LVAD’ AND ‘destination therapy’/exp OR ‘DT’ AND ‘heart failure’/exp OR ‘survival’	1375
Cochrane	“Left Ventricular Assist Device” OR “LVAD” AND “Destination Therapy” AND “Heart Failure” AND “Survival Rate”	402
Web of Science	TS = (left ventricular assist device OR LVAD) AND TS = (destination therapy OR DT) AND TS = (heart failure OR survival)	897
Scopus	TITLE-ABS-KEY (“Left Ventricular Assist Device” OR “LVAD”) AND (“Destination Therapy” OR “DT”) AND (“Heart Failure”)	1123

**Table 2:** Effect sizes and variances of the included studies.

Study	Effect Size	Variance or SE
Slaughter et al., 2009 [45]	0.42	0.57
Rogers et al., 2017 [40]	0.64	0.72
Goldstein et al., 2019 [46]	1.25	0.88
Kirklin et al., 2013 [47]	0.08	0.16
Krabatsch et al., 2017 [41]	0.54	0.34
Mehra et al., 2019 [42]	2.64	0.10
Najjar et al., 2014 [48]	1.18	0.60
Packer et al., 2016 [49]	0.66	1.15
Miller et al., 2007 [43]	0.19	0.38
Merzo et al., 2024 [50]	0.30	0.57
Slaughter et al., 2013 [44]	0.64	0.54
Pagani et al., 2009 [51]	1.00	0.89
Slaughter et al., 2009 [45]	0.82	0.70

**Table 3:** Random-effects model (k = 13).

	Estimate	se	Z	*p*	CI Lower Bound	CI Upper Bound
Intercept	0.848	0.277	3.07	0.002	0.306	1.390

Note: Tau^2^ estimator: restricted maximum-likelihood.

## Data Availability

Data are available upon request.
